# A Hybrid Closed-Loop Blood Glucose Control Algorithm with a Safety Limiter Based on Deep Reinforcement Learning and Model Predictive Control

**DOI:** 10.3390/bios16010047

**Published:** 2026-01-06

**Authors:** Shanyong Huang, Yusheng Fu, Shaowei Kong, Yuyang Liu, Jian Yan

**Affiliations:** School of Information and Communication Engineering, University of Electronic Science and Technology, Chengdu 611731, China; 202322011927@std.uestc.edu.cn (S.H.); yushengf@uestc.edu.cn (Y.F.); 202322010917@std.uestc.edu.cn (S.K.); 202352012017@std.uestc.edu.cn (Y.L.)

**Keywords:** artificial pancreas, blood glucose control, reinforcement learning, model predictive control

## Abstract

Due to the complexity of blood glucose dynamics and the high variability of the physiological structure of diabetic patients, implementing a safe and effective insulin dosage control algorithm to keep the blood glucose of diabetic patients within the normal range (70–180 mg/dL) is currently a challenging task in the field of diabetes treatment. Deep reinforcement learning (DRL) has proven its potential in diabetes treatment in previous work, thanks to its strong advantages in solving complex dynamic and uncertain problems. It can address the challenges faced by traditional control algorithms, such as the need for patients to manually estimate carbohydrate intake before meals, the requirement to establish complex dynamic models, and the need for professional prior knowledge. However, reinforcement learning is essentially a highly exploratory trial-and-error learning strategy, which is contrary to the high-safety requirements of clinical practice. Therefore, achieving safer control has always been a major challenge for the clinical application of DRL. This paper addresses this challenge by combining the advantages of DRL and the traditional control algorithm—model predictive control (MPC). Specifically, by using the blood glucose and insulin data generated during the interaction between DRL and patients in the learning process to learn a blood glucose prediction model, the problem of MPC needing to establish a patient’s blood glucose dynamic model is solved. Then, MPC is used for forward-looking prediction and simulation of blood glucose, and a safety controller is introduced to avoid unsafe actions, thus restricting DRL control to a safer range. Experiments on the UVA/Padova glucose kinetics simulator approved by the US Food and Drug Administration (FDA) show that the time proportion of adult patients within the healthy blood glucose range under the control of the model proposed in this paper reaches 72.51%, an increase of 2.54% compared with the baseline model, and the proportion of severe hyperglycemia and hypoglycemia events is not increased, taking an important step towards the safe control of blood glucose.

## 1. Introduction

Diabetes mellitus (DM) is a series of metabolic disorder syndromes caused by genetic factors, immune dysfunction, or other pathogenic factors acting on the human body, leading to the decline or even loss of islet function. Clinically, it is mainly characterized by hyperglycemia (normal blood glucose range: 70 mg/dL–180 mg/dL) [1]. According to the ability to effectively utilize insulin, diabetes can be divided into type 1 diabetes mellitus (T1DM) and type 2 diabetes mellitus (T2DM). According to the estimates of the International Diabetes Federation (IDF) Atlas, there were 537 million adults with diabetes worldwide in 2021. It is estimated that by 2045, the number of diabetes patients may reach 784 million, making it one of the fastest-growing health problems in the world in the 21st century [2]. Due to different pathogenesis, the treatment methods for the two types of diabetes patients are also different. Patients with type 2 diabetes (T2D) are mainly treated with oral hypoglycemic drugs, while for patients with type 1 diabetes (T1D) who have absolute insulin deficiency due to autoimmunity, blood glucose is mainly controlled by insulin injection [3], and this is also the patient group to be studied in this paper. The insulin infusion volume is crucial for the treatment of T1D patients. Insufficient insulin infusion will lead to an increase in the patient’s blood glucose. Prolonged hyperglycemia can cause complications such as vascular damage, retinopathy, renal failure, and neuropathy. In severe cases, it can also lead to ketoacidosis, causing vomiting, coma, and even threatening the patient’s life. On the other hand, excessive insulin infusion will cause the patient’s blood glucose level to be lower than the normal range. Prolonged hypoglycemia can damage brain cells, increase the risk of dementia, and increase the patient’s cardiac burden, leading to arrhythmia and myocardial ischemia. Severe hypoglycemia can be fatal within 6 h [4,5]. Therefore, real-time monitoring of blood glucose levels and accurate insulin infusion are crucial for T1D patients.

Currently, blood glucose monitoring methods have evolved from the traditional fingertip prick method, which can only provide single blood glucose measurement values, to continuous glucose monitoring (CGM) devices. These devices can provide continuous, comprehensive, and accurate blood glucose concentration information, helping patients better understand their blood glucose changes and significantly reducing the pain and the risk of needle-stick wound infection for patients [6,7,8].

**Figure 1:**
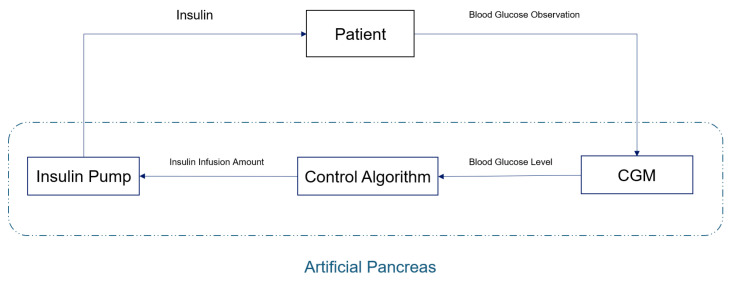
Schematic diagram of an artificial pancreas system.

With the development of CGM technology, blood glucose control methods have also evolved from the original manual calculation of insulin volume injection to the use of an artificial pancreas (AP) system to achieve continuous and automatic control of patients’ blood glucose. A schematic diagram of the artificial pancreas system is shown in Figure 1. It mainly consists of three parts: (1) a continuous glucose monitoring device (CGM) that can provide the patient’s current and historical blood glucose values; (2) a control algorithm that calculates the insulin infusion volume based on the patient’s blood glucose level; and (3) an insulin pump that injects insulin into the patient according to the algorithm. Among them, the control algorithm is the core of the AP system, as its quality directly determines the system’s performance [3,9,10].

Traditional control algorithms include PID control [11], model predictive control (MPC) [12], fuzzy logic control [13], etc. Among them, PID control and MPC have been approved by the U.S. Food and Drug Administration (FDA) and are applied in commercially available AP systems [14]. However, although PID control is easy to implement, it tends to overestimate postprandial insulin doses and struggles to integrate other blood glucose-influencing factors, such as patient exercise data and insulin activity [15,16]. While MPC is more adaptable, its performance depends on the established blood glucose dynamic model. If a linear or other simplified model of glucose kinetics is used, it may fail to capture all the dynamic characteristics of blood glucose changes. If a more complex physiological model is to be established, extremely professional biomedical knowledge is required, making it more difficult to implement [17,18]. The blood glucose regulation system is a complex non-linear dynamic system, which has high inter-individual and intra-individual variability, as well as uncertainties and disturbances related to diet, exercise and daily events [19]. In addition, subcutaneous drug administration causes a delay in insulin action, and changes in the patient’s insulin sensitivity and sensor lag delay the effect of insulin, resulting in large fluctuations in post-meal blood glucose and increasing the complexity of blood glucose control. All the above control strategies are affected by these characteristics [20,21,22]. More importantly, these systems require patients to manually estimate carbohydrates before meals. This not only increases patient cognitive burden but may also inadvertently lead to erroneous algorithmic decisions caused by inaccurate carbohydrate intake estimation, which may compromise patient health [23,24].

Reinforcement learning (RL) has achieved success in many complex control tasks, such as playing games and robot control [25,26]. In RL algorithms, an agent interacts with its environment to achieve specified goals. When the agent takes an action, it causes a state transition in the environment. Meanwhile, the predefined reward function evaluates the feedback signal (reward) related to the pursued goal in this state and returns it to the agent. The agent uses its experience (states, actions, rewards) to learn a control strategy to maximize the cumulative reward. With the development of deep learning (DL), deep reinforcement learning (DRL), which applies DL networks and modules to the RL framework, provides more flexibility in implementing policy networks and value networks [27]. DRL does not require prior physiological knowledge to build a complex blood glucose dynamics model. Instead, it learns strategies through exploration and trial-and-error, and has great potential in achieving fully automated closed-loop control of artificial pancreas [20,28].

**Figure 2:**
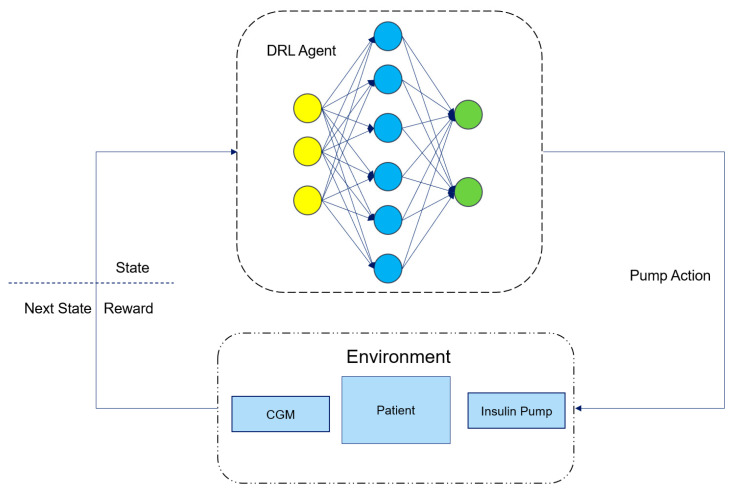
Schematic diagram of an artificial pancreas system based on DRL.

Figure 2 is a schematic diagram of the working principle of an artificial pancreas system based on deep reinforcement learning. Here, the DRL Agent is an RL algorithm implemented using DL network modules. It calculates the insulin dose required by the patient based on the current state provided by the environment, i.e., the patient and the connected insulin pump and CGM sensor, and interacts with the patient through the insulin pump to inject insulin into the patient’s body. After the DRL Agent takes an action, the environment monitors and returns the patient’s next state through the CGM and calculates the reward for the DRL Agent’s current action based on the next state. The DRL Agent then adjusts its strategy according to the returned next state and reward to keep the patient’s blood glucose level within a healthy range.

The application of RL in controlling the blood glucose levels of T1D patients can be traced back to 2012. Daskalaki et al. [29] studied the use of the actor–critic method in blood glucose control. However, due to the limitations of the available simulators at that time, this task was limited to daily updates of the control strategy. With the release of the FDA-approved UVA/Padova T1D simulator Simglucose [30], the application of RL in the control of artificial pancreas has attracted more and more attention from researchers. But many early studies were based on the Q-learning method. Although it alleviated the impacts of insulin action delay, changes in patients’ insulin sensitivity, and sensor lag delay in the blood glucose control task, it required patients to manually estimate carbohydrate intake and provide meal announcements, which imposed a cognitive burden on patients [31,32,33].

In recent years, many advanced DRL algorithms have been proposed for blood glucose control tasks and to eliminate the influence of carbohydrate overload and control estimation. Fox [34] et al. compared the performance of DRL and non-RL methods through a series of experiments. The DRL method was implemented using Soft Actor–Critic. In more than 2.1 million hours of data from 30 simulated patients, the SAC-based DRL method successfully reduced the median blood glucose risk by nearly 50% and the total hypoglycemia time by 99.8%. Moreover, it did not require patients to manually estimate carbohydrate intake, allowing patients to manage their blood glucose levels without professional knowledge. Lee [35] et al. developed a bio-inspired RL design method for automatic insulin infusion based on PPO. This method has a reward function that implies a time-steady-state goal and a discount factor that reflects individual-specific pharmacological characteristics. Experiments showed that the AP algorithm based on the bio-inspired RL method can achieve fully automatic blood glucose control without announced meal intake. Federico [36] et al. developed a new type of DRL controller for the autonomous blood glucose regulation of patients with T1D. This controller is based on the DDPG algorithm and overcomes the limitations of standard clinical practice and classical model-based control techniques in terms of control workload and computational efficiency for real-time applications. However, these algorithms still lack safety-oriented design considerations and are not yet ready for clinical translation.

While deep reinforcement learning (DRL) offers distinct advantages over traditional control methods like PID control and model predictive control (MPC), its clinical translation faces critical challenges. As an exploration-driven, trial-and-error learning paradigm, DRL inherently requires iterative refinement to develop optimal policies, leading to suboptimal performance during early training phases [37]. In non-clinical domains (e.g., gaming, robotics), such transient errors are tolerable due to minimal associated costs. However, in clinical settings, even minor decision-making errors may pose significant risks to patient safety, making unconstrained trial-and-error learning infeasible. Additionally, DRL’s data-intensive nature presents a practical barrier. Previous studies have shown that DRL models often require training on simulated patient data spanning years or even decades to achieve robust control performance [34,38]. Translating this to clinical practice would necessitate prolonged deployment of unvalidated controllers, which is ethically and practically unacceptable. Thus, key challenges for DRL in clinical applications include ensuring safety during learning, enhancing training efficiency, and reducing data requirements.

Many researchers have also dedicated themselves to the safety research of DRL in blood glucose management, and most of them are based on a hybrid control strategy that combines model-free and model-based approaches. Reference [39] proposed a new hybrid control strategy for the AP system called HyCPAP to leverage the advantages of MPC and DRL strategies and compensate for their respective shortcomings. By combining the MPC strategy with the overall DRL strategy, HyCPAP provides personalization and adaptability while ensuring safety and stability. However, the insulin–glucose dynamic model used in its MPC is a discrete-time linear time-invariant model, which cannot effectively capture all the dynamic characteristics of blood glucose changes. Reference [40] combined PID control with the DRL strategy. It adopted PID control in the early stage of DRL strategy training and gradually reduced the weight of PID control as the training progressed, gradually transitioning to DRL control. The results were comparable to those using PID control, and it successfully captured glucose dynamics with little prior knowledge. However, the input of its model still included carbohydrate intake, so it was not a fully closed-loop control. Chirath [41] et al. proposed a modular DRL method, introducing an auxiliary learning stage and a planning stage to optimize the DRL strategy in the short term, effectively reducing the patient’s hypoglycemia time. However, in the planning stage, it only selected the action with the maximum cumulative reward value during the simulation process and did not use the prediction model to limit potentially dangerous actions. Reference [42] adopted ten techniques to enhance the PPO algorithm and introduced a dual safety mechanism of “active guidance + reactive correction” to reduce the risks of hyperglycemia and hypoglycemia and prevent emergencies. However, its correction controller performs reactive correction based on the blood glucose monitored by the current CGM and cannot play a preventive role. Harry [43] et al. evaluated offline RL without potentially dangerous patient interactions during training. The training data for offline RL were collected through PID control. This work shows that when training with less than one-tenth of the total training samples required for online RL to achieve stable performance, offline RL can significantly increase the time within the healthy blood glucose range, from 61.6 ± 0.3% to 65.3 ± 0.5%.

Combining the advantages of previous research results, we proposed a hybrid control strategy based on DRL and MPC, and introduced a safety controller in combination with clinical experience. Compared with the literature [39], this study has two core technical differences in the design of the MPC controller and the collaboration mechanism. First, at the level of constructing the blood glucose dynamic model, we abandoned the discrete-time linear time-invariant model framework that relies on patients’ physiological parameters. Instead, we used the real-time interaction data between DRL and patients to drive model learning, and introduced the Informer network with outstanding long-time series modeling ability for feature extraction, realizing the adaptive construction of a personalized blood glucose dynamic model without prior knowledge. Second, in the collaboration strategy between MPC and DRL, we generated optimal action samples through multiple rounds of simulation optimization of MPC, which were used as guiding learning signals for the DRL model to accelerate strategy convergence. However, the literature [39] adopted a multi-DRL output consistency voting mechanism and only introduced the MPC output as a compromise solution when there were strategy disagreements. During the simulation control process of MPC, we predict the blood glucose value after taking candidate actions through the prediction module. When the predicted blood glucose value is lower than 54 mg/dL, the candidate action sequence containing this action is marked as unsafe. Finally, the action with the maximum cumulative reward is selected from the safe actions as the next action of DRL. If all actions are unsafe, the action of the safety controller is taken, so as to adjust the patient’s blood glucose to the healthy range in the short term. We expect that through such double-safety control, a safer fully automatic closed-loop control of the blood glucose level of T1D patients can be appropriately achieved.

## 2. Materials and Methods

### 2.1. Problem Definition of Blood Glucose Control in the Reinforcement Learning Task

Since the glucose level data monitored by the CGM sensor is noisy and it is difficult to observe other state information, such as insulin level, diet, exercise information, etc., the blood glucose control problem of the artificial pancreas system is a partially observable Markov decision process (POMDP). A POMDP is usually represented by a tuple (*T*, *S*, *A*, *P*, *O*, *R*). Here, *t* ∈ *T* represents the true state of the current environment, *s* ∈ *S* represents the state observed by the observation function *O*, and *a* ∈ *A* represents the action taken. The state transition function *P*: (*t*, *a*) -> *t*′ describes the dynamics of the system, which means that when the reinforcement learning agent takes action a in the current environment state t, the environment transitions to the next state *t*′. The observation function *O*: *t* -> *s* maps the true state t of the environment to the observed state s. The reward function *R*: (*s*, *a*) -> *r* represents the reward *r* ∈ *R* obtained by the agent after performing action a in the state s it observes. In reinforcement learning, the agent’s task is to try different actions in different states to interact with the environment and learn from the rewards obtained to maximize the cumulative rewards. In the blood glucose control task, our goal is to train an agent to control the actions of the insulin pump by learning the strategy $\pi (a_{n}|s_{n})$ based on the current patient’s blood glucose level and the infused insulin dose information, so as to maximize the average reward:

(1) $$\bar{R}=\frac{1}{N}\sum_{n=1}^{N}E[r_{n}|\pi (a_{n}|s_{n})],$$

True state space T: The patient’s true physiological state and environmental factors, such as blood glucose and insulin levels, dietary intake, exercise volume, etc. The state space is complete. However, since the closed-loop system of the artificial pancreas cannot directly observe all of the patient’s states, it is partially observable.

Observation state space S: The patient’s observed state refers to the patient’s blood glucose value g measured by the CGM sensor and the insulin dose $i_{t}$ injected by the insulin pump. Usually, we use the observed k historical data as the patient’s current observed state input to the system. Therefore, the patient’s state can be defined as:

(2) $$s_{t}\left(g_{t-k},g_{t-k+1},\cdots ,g_{t-1},g_{t},i_{t-k},i_{t-k+1},\cdots ,i_{t-1},i_{t}\right),$$

Action space A: To provide more flexibility for the control strategy learned by the reinforcement learning agent, we define the space of the action $a_{t}$ output by it as a continuous space and limit it within the range of [−1, 1]. Then, we convert the action $a_{t}$ output by the RL agent into the insulin infusion rate $I_{t}$ (U/min) of the insulin pump through the nonlinear transformation Formula (3) proposed in Reference [44].

(3) $$I_{t}=I_{\max}e^{\tau (a_{t}-1)},$$

Among them, $I_{\max}$ represents the maximum rate of insulin infusion per minute, which is usually set to 5, while τ is set to 4.0. $a_{t}$ ∈ [−1,1] represents the action output by the RL agent at time t.

Reward function R: The reward function is the core driving force of reinforcement learning. It defines the goals and behavioral guidelines of the agent, and directly determines the learning direction, efficiency, and performance of the agent. Reference [33] defines a blood glucose risk index (Equation (4)) to measure the risk of the patient’s current blood glucose level $g_{t}$ based on the clinical low blood glucose risk (LBGI) and high blood glucose risk (HGBI).

(4) $$risk(g_{t})=10\times {\left(1.509\times \ln{\left(g_{t}\right)}^{1.084}-5.381\right)}^{2},$$

**Figure 3:**
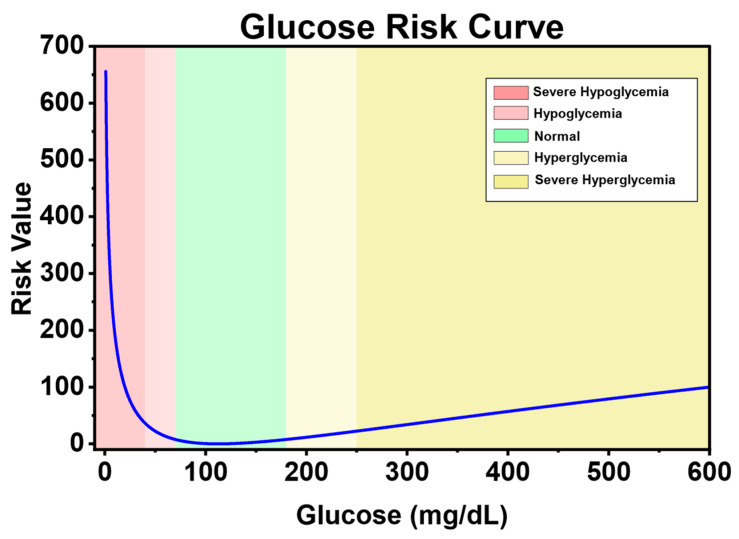
Blood glucose risk index curve.

Figure 3 shows the change in the risk index with blood glucose level. It can be seen that the blood glucose risk gradually increases as it deviates from the healthy range (70–180 mg/dL). Moreover, hypoglycemia has a higher risk value than hyperglycemia. This is because the harm of hyperglycemia is more of a chronic cumulative nature, while the harm of hypoglycemia is more acutely fatal. Therefore, when blood glucose is in the state of hypoglycemia and severe hypoglycemia, it has a higher risk.

**Figure 4:**
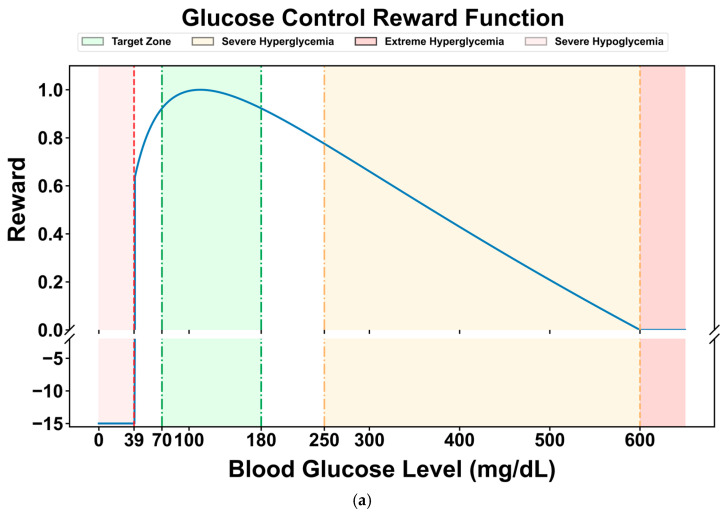

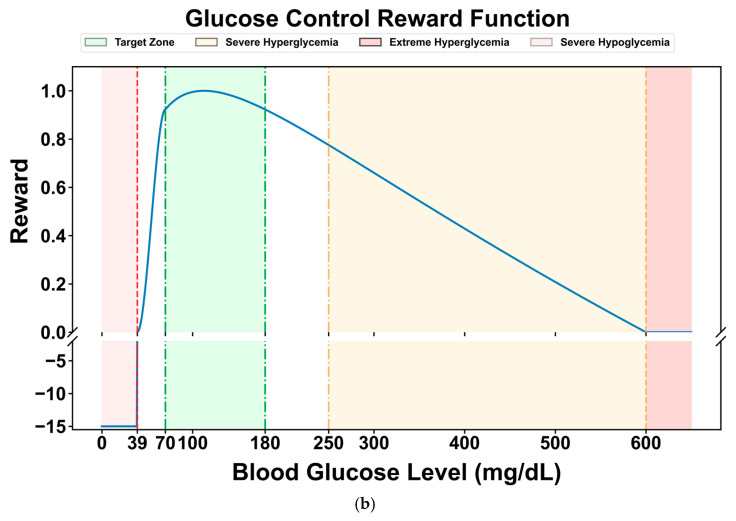
Comparison of two reward curves: (**a**) reward function curve proposed in reference [45]; (**b**) the reward function curve used in this paper.

Many researchers have designed reward functions for reinforcement learning in blood glucose control tasks based on the blood glucose risk index. Reference [45] proposed a reward function (Equation (5)), which normalizes the blood glucose risk index to the range of [−1, 0] and takes its negative value as the reward for the current blood glucose level. For severe hypoglycemia events when the blood glucose level is less than 40 mg/dL, a penalty term of −15 is set to punish hypoglycemic events that pose a serious threat to the patient’s life and guide the agent to avoid severe hypoglycemia events. Its graph is shown in Figure 4a.

(5) $$r_{t}(s_{t},a_{t})=\left\{\begin{array}{l} -15,g_{t+1}<=40\mathrm{mg}/\mathrm{dL}\\ -risk(g_{t+1}),\mathrm{else} \end{array}\right.,$$

Compared with the limitation of the original reward function, which only sets a single penalty term in the severe hypoglycemia range (<40 mg/dL), this study introduces cubic spline smoothing for the hypoglycemic state (40–70 mg/dL). The original function still has relatively high reward values in this range, which may lead to insufficient sensitivity of the DRL strategy to mild hypoglycemic risks. By using a cubic spline function to perform continuous non-linear smooth attenuation of the reward values in this range (gradually decreasing from the baseline reward value at 70 mg/dL to 0 at 40 mg/dL), a dual optimization of refined risk gradient and improved strategy stability can be achieved. The former constructs a continuous penalty gradient from “mild hypoglycemia → severe hypoglycemia”, enabling the DRL model to more accurately perceive the degree of blood glucose deviation and avoid the “step-like” lag in responding to hypoglycemic risks. The latter reduces the reward jumps during state transitions by smoothing the reward curve, thereby reducing the oscillations in strategy updates and enhancing the stability of the learning dynamics, especially in the critical range with frequent blood glucose fluctuations (e.g., 50–60 mg/dL).

The modified reward function is shown in Figure 4b. It maintains a high-reward plateau in the normal blood glucose range (70–180 mg/dL), achieves a continuous attenuation of the reward value from the baseline value to 0 through cubic splines in the hypoglycemic range (40–70 mg/dL), and maintains a strong penalty in the severe hypoglycemic range (<40 mg/dL), forming a hierarchical reward mechanism of “protecting normal blood glucose → gradually avoiding hypoglycemia → strictly punishing severe hypoglycemia”.

### 2.2. Model Design

As a powerful artificial intelligence technology, deep reinforcement learning accumulates experience through interaction with the environment and gradually improves the quality of decision-making. It provides the core advantages of autonomous learning and optimization in complex and uncertain environments. This paper proposes a hybrid control model based on deep reinforcement learning and model predictive control, and introduces a safety controller. The aim is to completely eliminate the patient’s manual estimation of carbohydrates, improve the decision-making safety of the RL model, achieve safe and fully automatic closed-loop control of the artificial pancreas, and promote the application of deep reinforcement learning in diabetes management.

**Figure 5:**
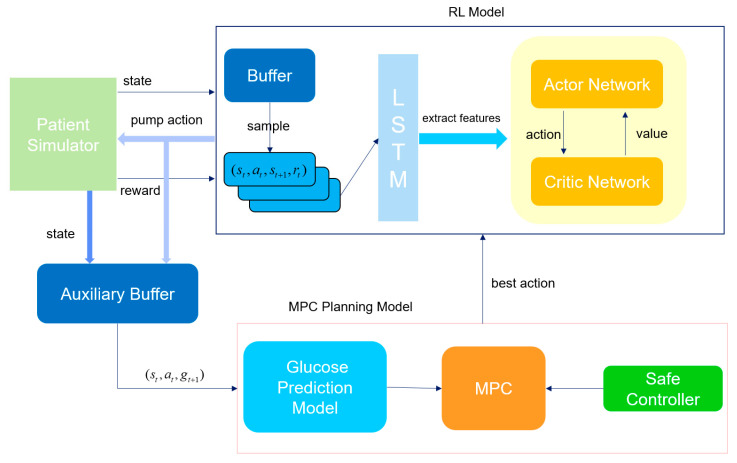
Framework diagram of the hybrid control model with a safety controller.

The overall schematic diagram of the model proposed in this paper is shown in Figure 5. First, the reinforcement learning model (RL agent) interacts with the patient simulator, which simulates the patient’s physiological state (such as historical blood glucose values and historical medication records) and the response to actions. In each round of interaction, the RL agent receives the sequential data of the current state output by the simulator, processes it through the long-short-term memory network (LSTM) of the feature extraction module to capture the time-dependent information in the state (such as the changing trend of blood glucose), and generates a hidden state. This hidden state is input into both the Actor network and the Critic network. As the policy network, the Actor network outputs specific actions based on the hidden state. This action is passed to the simulator, which updates the patient’s state (blood glucose value at the next moment) according to the action and returns the corresponding reward. These interaction data (state, action, reward, next state) are stored in the buffer container (experience replay pool) for subsequent batch learning to break the time correlation of the data and improve the training stability. After a round of interaction ends, batch data are randomly sampled from the buffer to update the Actor and Critic networks. For the Critic network (value network), its task is to estimate the value of the state or state-action, that is, the expected total reward that can be obtained in the future after taking a certain action in the current state. Specifically, the Critic predicts a Q-value (state-action value) based on the hidden state of the current state and the action output by the Actor, and then calculates the prediction error (TD error) using the immediate reward returned by the simulator plus the discounted Q-value of the next state (TD target). The parameters of the Critic are updated by minimizing this error through back-propagation to make its value estimation more accurate. The update of the Actor network depends on the value estimation of the Critic. The goal of the Actor is to maximize the expected reward, so it needs to adjust the policy according to the advantage signal given by the Critic. Through such coordinated updates, the Actor gradually learns to output better actions, and the Critic continuously improves the accuracy of value estimation. The two optimize together to enable the RL agent to better control the patient’s blood glucose state and balance the risks of hypoglycemia and hyperglycemia.

In the iterative interaction between the main RL process and the patient simulator, the auxiliary buffer acts as a “data bridge”, continuously collecting state-action-state transition samples generated during the reinforcement learning update process. Specifically, these include: the patient state currently perceived by the RL agent (time-series data of historical blood glucose values and insulin doses), the insulin pump action output by the Actor network, and the real blood glucose value at the next moment simulated by the simulator based on this action. These data are specifically used to accumulate supervised learning samples for the training of the blood glucose prediction model, with the goal of enabling the prediction model to learn to “accurately predict the blood glucose change at the next moment based on the current state and the upcoming action”. The working logic of the auxiliary buffer follows the “batch collection-trigger training” mode: when the number of samples in the buffer reaches the preset threshold, the system automatically initiates the learning process of the blood glucose prediction model. At this time, the data in the buffer will be taken out in batches and organized according to the supervised learning paradigm of “input-label”. The input layer integrates the time-series features of the current state and the numerical features of the RL action, while the label layer is the real blood glucose value at the next moment returned by the simulator. The prediction model generates the predicted blood glucose value at the next moment through forward propagation, then calculates the error between the predicted value and the real value, and adjusts the model parameters through the error backpropagation algorithm to minimize the prediction error. In essence, this process allows the prediction model to “learn” the law of how the patient state and insulin action jointly affect blood glucose changes.

When the prediction accuracy of the blood glucose prediction model reaches the preset threshold (RMSE drops to 15 mg/dL), the system will activate the MPC (Model Predictive Control) module. Through multi-trajectory simulation and safety screening, the action output of the RL (Reinforcement Learning) agent will be optimized. The specific process is as follows: First, based on the patient’s current physiological state, the MPC generates multi-step simulations of multiple future blood glucose change trajectories (each trajectory corresponds to a different insulin pump action sequence). For each action at each step of each trajectory, the blood glucose prediction module is called to calculate the corresponding predicted blood glucose value at the next moment. If the predicted blood glucose at a certain step is lower than 70 mg/dL (hypoglycemia threshold) or higher than 250 mg/dL (severe hyperglycemia threshold), the entire trajectory is marked as “unsafe”. After the simulation is completed, the system only retains the safe trajectories (the predicted blood glucose level at all steps is within the safe range) and calculates the cumulative reward value for each safe trajectory. Finally, the safe trajectory with the maximum cumulative reward value is selected, and its first-step action is taken as the optimal action that the RL agent should execute in the current state. If all trajectories are marked as unsafe (i.e., any action sequence may lead to hypoglycemia or severe hyperglycemia), the safety controller will immediately intervene: it calculates an insulin dosage adjustment coefficient based on the patient’s current blood glucose value and recent change trend, and corrects the action output by the RL to limit it to a more conservative safe range. The core goal of this mechanism is to quickly resolve short-term blood glucose risks (such as impending hypoglycemia) and avoid ignoring emergencies due to the “long-term optimization” of the RL strategy. We hope that through such a dual-safety control mechanism, we can effectively avoid the unsafe actions that the RL may generate during the learning process and affect the patient’s health.

The closed-loop control model proposed in this paper integrates the model-free reinforcement learning method and the model-based MPC method. It combines the advantages of deep learning in automatically extracting features and reinforcement learning in continuous decision-making to construct an end-to-end closed-loop blood glucose control system. The entire process from input to output is completed by the control model without human intervention or complex intermediate steps. This end-to-end deep reinforcement learning method further improves the blood glucose control effect for diabetic patients, making the control strategy more intelligent and personalized.

#### 2.2.1. Reinforcement Learning Model

In RL algorithms, we considered three current advanced RL algorithms: SAC, DDPG, and PPO. All three algorithms adopt the Actor–Critic architecture, but there are slight differences in specific network implementation and update methods.

Specifically, as a representative of the deterministic policy gradient algorithm, the Actor network of DDPG uses a deterministic structure, directly outputting specific action values in the continuous action space. This design simplifies the action generation process, but due to the lack of randomness, it may lead to insufficient exploration. The corresponding Critic network is a single Q-network, which takes the state and action as inputs and outputs the state-action value Q(s,a) to evaluate the value of the current action and provide signals for the gradient update of the Actor. To stabilize the training, DDPG introduces dual target networks (target Actor and target Critic), generating target actions and target Q-values through soft updates to avoid the interference of current network fluctuations on the training. During the update, the update target of the Actor network is to maximize the Q-value evaluation of the current action by the Critic network. That is, the policy is adjusted through the feedback of the Critic to make the generated actions better. The update formula for its network parameters θ is as follows:

(6) $$\theta \leftarrow \theta +\alpha \cdot {▽}_{\theta }Q_{\varpi }(s,\mu_{\theta }(s)),$$

Among them, $\mu_{\theta }(s)$ is the deterministic action output by the Actor network, and $Q_{w}(s,\mu_{\theta }(s))$ is the state-action value output by the Critic. α is the learning rate of the Actor network. In the update of the Critic network, all three use the TD error to calculate the network loss. The TD error is the deviation between the current state value estimate and the “immediate reward + future state value estimate”. Its formula is:

(7) $$\delta =r_{t}+\gamma V(s_{t+1})-V(s_{t}),$$

For DDPG, its Critic network estimates the state-action value function Q(s,a), and its update target is the TD target generated by the target network:

(8) $$y_{t}=r_{t}+\gamma Q_{target}(s_{t+1},\mu_{target}(s_{t+1})),$$

The loss function of the Critic network is as follows:

(9) $$L=E[(Q(s_{t},a_{t})-y_{t}{)}^{2}],$$

PPO focuses on stochastic policy optimization. Its Actor network outputs the parameters of the action distribution, and actions are obtained by sampling the distribution. The randomness enhances the exploration ability. The Critic network is a single V network that only takes the state as input and outputs the state value V(s), which is used to estimate the baseline value of the state. It calculates the target for policy update in combination with the Generalized Advantage Estimation (GAE). Different from DDPG, PPO does not require a target network. Instead, it limits the policy update amplitude through the “clipped advantage function”, which not only ensures the training stability but also simplifies the network structure. It is suitable for a wide range of tasks in both discrete and continuous action spaces. The Actor network of PPO outputs a stochastic policy π(a|s), and the goal of its update is to maximize the policy performance under the premise of constraining the policy change amplitude, avoiding training instability caused by sudden policy changes. The specific update formula is as follows:

(10) $$\theta \leftarrow \theta +\alpha \cdot {▽}_{\theta }E[\min(\frac{\pi_{\theta }(a|s)}{\pi_{\theta_{old}}(a|s)}\cdot A(s,a),clip(\frac{\pi_{\theta }(a|s)}{\pi_{\theta_{old}}(a|s)},1-\varepsilon ,1+\varepsilon )\cdot A(s,a)],$$

Among them, $\pi_{\theta }(a|s)$ is the policy of the current Actor network, which outputs the parameters of the action distribution, namely the mean and variance of the Gaussian distribution; while $\pi_{\theta_{old}}(a|s)$ is the old policy before the update, used to calculate the ratio of policy change; A(s,a) is the advantage function, usually estimated by GAE, which measures the superiority or inferiority of action a relative to the average action; ϵ is the truncation parameter, which limits the amplitude of policy change. The loss function of the Critic network in PPO is as follows:

(11) $$L=\frac{1}{2}E[{\left(G_{t}-V(s_{t})\right)}^{2}],$$

Among them, $V(s_{t})$ is the state value output by the Critic network, and Gt is the weighted sum of the TD errors, which is calculated as follows:

(12) $$G_{t}=r_{t}+\gamma V(s_{t+1})+\gamma^{2}V(s_{t+2})+\ldots +\gamma^{T-t}V(s_{T}),$$

As a typical algorithm of maximum entropy reinforcement learning, the Actor network of SAC also has a stochastic structure, but it uses the reparameterization trick to enable efficient gradient transmission. The Critic network uses a double Q-network (Q1 and Q2), both of which take the state and action as inputs and output two Q-values. During training, the minimum value is taken as the target Q-value, which effectively alleviates the overestimation bias of Q-values and improves the accuracy of value evaluation. In addition, SAC introduces a temperature parameter α. By balancing the expected return and the policy entropy, it encourages more comprehensive exploration, making it show stronger adaptability in continuous control tasks. In terms of the target network, SAC only uses a double target Critic network (target Q1′ and Q2′) and keeps the target value stable through soft updates. The update of the Actor network depends on the output of the current Critic, and there is no need for a target Actor. The Actor network of SAC also outputs a stochastic policy $\pi_{\theta }(a|s)$. Its update goal is to maximize the expected return with entropy regularization; that is, while pursuing high returns, it maintains the randomness (entropy) of the policy to enhance the exploration ability. Therefore, its update formula is:

(13) $$\theta \leftarrow \theta +\alpha \cdot {▽}_{\theta }E[Q_{\phi }(s,a)-\lambda \log\pi_{\theta }(a|s)],$$

Among them, $Q_{\phi }(s,a)$ is the state-action value output by taking the minimum value of the double Q-networks of the Critic network, and the minimum value of the double Q-networks is taken to reduce the overestimation bias; is the entropy of the policy, which is used to measure the randomness of actions, and $\log\pi_{\theta }(a|s)$ is the temperature parameter used to balance “reward maximization” and “entropy maximization”. For the Critic network, SAC uses double Q-networks to estimate the state-action value function, and its update target is the TD target with entropy regularization to balance rewards and exploration:

(14) $$y_{t}=r_{t}+\alpha E_{\pi_{target}(a\prime |s_{t+1})}[\min(Q_{1}^{target}(s_{t+1},a\prime ),Q_{2}^{target}(s_{t+1},a\prime ))-\lambda \log\pi_{target}(a\prime |s_{t+1})],$$

The loss function is:

(15) $$L=\frac{1}{2}E[{\left(Q_{1}(s_{t},a_{t})-y_{t}\right)}^{2}+{\left(Q_{2}(s_{t},a_{t})-y_{t}\right)}^{2}],$$

**Figure 6:**
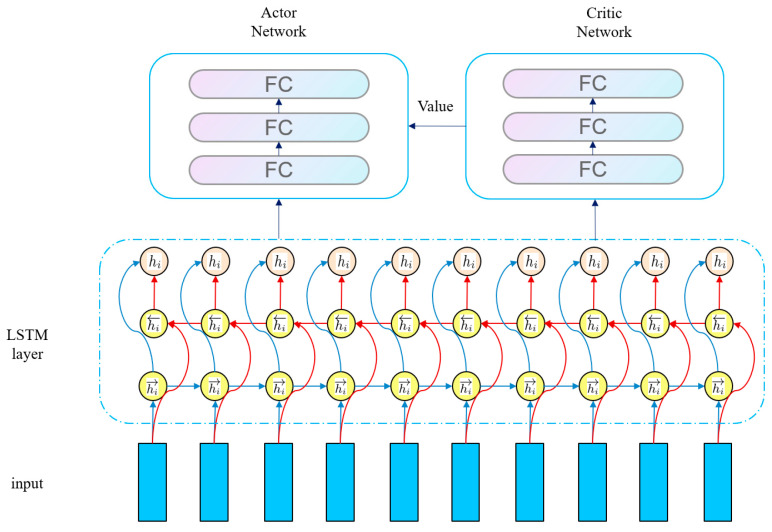
Network structure of RL algorithm.

All three RL algorithms are based on the Actor–Critic (AC) architecture and use a shared Long Short-Term Memory network (LSTM) as the feature extraction module. When extracting features from the original state, they share the same set of LSTM parameters. The extracted sequential features are then input into the policy head of the Actor and the value head of the Critic, respectively. LSTM is selected because its gating mechanism can effectively capture the long-term temporal dependencies between states, which is suitable for the requirements of sequential states, partially observable environments (POMDP), and dynamic environments in RL tasks. It is more suitable for processing sequential data than ordinary MLP or CNN. The shared module is based on the high consistency of feature requirements between the Actor and the Critic in the AC architecture. It can not only reduce overfitting and improve generalization ability through parameter sharing but also ensure feature consistency to avoid the mismatch between decision-making and evaluation. Moreover, it can reduce redundant calculations and improve training efficiency. This design precisely adapts to the characteristics of the three algorithms (continuous action control of DDPG, trajectory sampling stability of PPO, and exploration–reward balance of SAC). In essence, it deeply combines the sequence modeling advantages of LSTM with the feature requirements of the AC architecture, laying the foundation for subsequent policy generation and value evaluation. After constructing the RL model, we will evaluate the performance of these three RL algorithms and select the optimal one for subsequent experiments. The overall network structure of the RL algorithms is shown in the following Figure 6.

#### 2.2.2. Blood Glucose Prediction Model

As classic representatives of model-free deep reinforcement learning (DRL), SAC, DDPG, and PPO all follow the core logic of “learning from interaction”—they do not rely on an explicit mathematical model of the environment. Instead, they completely collect experience data through real-time interaction between the agent and the environment to drive the neural network to optimize the strategy. However, this characteristic also brings the inherent problem of low sample utilization efficiency: in order to learn an effective strategy to handle complex scenarios, the algorithm often needs to conduct thousands or even millions of interaction attempts. In fields such as healthcare, where high requirements for safety and real-time performance are placed, this problem is particularly prominent: a long training time means that patients have to bear more unknown risks, and the low sample efficiency also limits the practical application of DRL in clinical scenarios.

To address this bottleneck, we propose a hybrid framework of “model-free strategy optimization + model-assisted decision-making”: in addition to the main DRL strategy module, an auxiliary learning module is integrated. By introducing an auxiliary task of the target object’s blood glucose dynamic model, efficient utilization of experience data and precise optimization of the strategy are achieved. Specifically: the auxiliary module takes the patient’s historical blood glucose data and insulin infusion as inputs, and uses a time-series prediction model to learn the dynamic law of blood glucose levels changing with the input actions (insulin injection) and the patient’s historical blood glucose; the trained blood glucose dynamic model is embedded in the model predictive control (MPC) framework, and serves as a “virtual environment” to conduct multi-step rolling simulations of the candidate actions output by the DRL strategy. Through this hybrid framework, the exploration ability of model-free DRL (it can adapt to unknown changes without prior knowledge of the environmental model) is combined with the sample efficiency of model assistance (reducing the number of actual interactions through virtual simulations), which avoids the agent from trying these actions in actual interactions and saves a large amount of useless exploration time.

**Figure 7:**
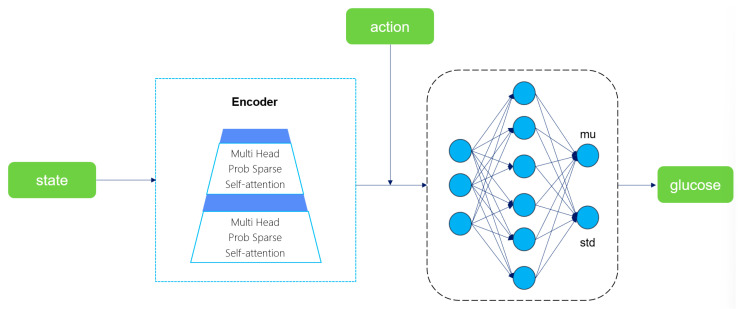
Schematic diagram of the blood glucose prediction model structure.

We designed an auxiliary buffer to accumulate the triplet data generated during the DRL process. When the buffer is full, the blood glucose prediction model training is initiated. This model takes the current state and action as inputs, encodes the time-series features through the Encoder module of Informer [46], and then outputs the mean and variance of blood glucose prediction via the fully connected layer. Finally, the next-step blood glucose prediction value is generated by sampling from the Gaussian distribution. The optimization goal is to minimize the error between the predicted value and the real value. The choice of Informer’s Encoder instead of the complete model or other time-series models is mainly based on three core advantages: First, through the Prob Sparse Self-Attention mechanism, Informer can reduce the computational complexity of long sequence processing while maintaining the ability to capture the key long-term dependencies for blood glucose prediction. Second, the Encoder module focuses on feature extraction, and its lightweight design meets the real-time requirements of DRL, avoiding the redundant computation of the complete model. Third, the output mean and variance support DRL to perceive the prediction uncertainty, facilitating robust decision-making. The overall framework of the blood glucose prediction model is shown in Figure 7.

#### 2.2.3. MPC Simulation and Safety Controller

When the prediction error of the blood glucose prediction model drops below 15 mg/dL, the system will initiate the MPC simulation process, optimizing the action output of the reinforcement learning agent through multiple multi-step simulations. Specifically, the RL agent first generates a next-step action suggestion based on the patient’s current state. The blood glucose prediction model then predicts the blood glucose level at the next moment after the action is taken. This predicted value is not the end but serves as a new state input, continuously updating the state space and calculating the reward for each step. This cycle continues until the preset number of simulation steps (6 steps, 30 min) is completed, and the action, state, and reward are recorded at each step. After 30 simulations are completed, the system will select the trajectory with the maximum cumulative reward from all trajectories and use the first-step action of this trajectory as the final output of the RL agent in the current state. This approach allows the RL agent to balance short-term rewards and long-term stability through multi-step simulations, avoiding “short-sighted” decisions.

To ensure the safety of RL actions, we designed a “dual-restriction” safety mechanism. The first is “trajectory filtering”: during the simulation process, the predicted blood glucose value is monitored in real time. If the predicted blood glucose of a certain trajectory is lower than 54 mg/dL (the severe hypoglycemia threshold, which may endanger life), it is immediately marked as “unsafe”. When selecting the optimal action in the end, only the action with the maximum cumulative reward is selected from the “safe trajectories”, which is equivalent to setting a “safety threshold” for the RL agent and eliminating the selection of dangerous actions. The second is “extreme situation handling”: if all trajectories are unsafe, the safety controller will take over. Its core is to calculate the insulin adjustment coefficient, taking into account three major factors: time characteristics, blood glucose status, and blood glucose trend. The calculated coefficient will modify the output of the RL agent. This design can quickly avoid the risk of severe hypoglycemia in the short term, providing a “last line of defense” for patients.

Referring to the literature [42], the calculation formula for the insulin adjustment coefficient of the safety controller is:

(16) $$c_{f}=coef\times \frac{obs[0]}{112.517}\times {\left(\frac{obs[0]}{obs[1]}\right)}^{2},$$

Among them, coef is a coefficient introduced to alleviate the problem of nocturnal hypoglycemia. Its value refers to the suggestions in the medical guidelines [47] and is set to 0.8 from 20:00 to 23:00 and 0.6 from 0:00 to 3:00. obs[0] and obs[1] are the blood glucose values at the current moment and the previous moment, respectively. 112.517 is the blood glucose value corresponding to a blood glucose risk index of 0. Therefore, $\frac{obs[0]}{112.517}$ represents the degree to which the current blood glucose deviates from the target blood glucose, while $\frac{obs[0]}{obs[1]}$ represents the changing trend of blood glucose. When the current blood glucose value is greater than 112.517 and the blood glucose is in an upward state, the insulin dose needs to be increased, and at this time $\frac{obs[0]}{112.517}\times {\left(\frac{obs[0]}{obs[1]}\right)}^{2}$ is greater than 1. When the blood glucose value is less than the target blood glucose value and is in a downward state, the insulin dose needs to be reduced, and at this time $\frac{obs[0]}{112.517}\times {\left(\frac{obs[0]}{obs[1]}\right)}^{2}$ is less than 1, which is in line with expectations. In particular, if obs[0] < 54 mg/dL, that is, the patient is in a state of severe hypoglycemia, the coefficient will be directly set to 0 to prevent the patient from being in life-danger.

During the MPC simulation phase, the goal of the RL agent’s learning is to minimize the deviation between the current policy $\pi (a_{t}|s_{t})$ and the optimal policy $\pi (a_{t}^{*}|s_{t})$ as much as possible. Therefore, its loss function is:

(17) $$L_{loss}(\theta^{\pi })=E[-\log(\pi (a_{t}^{*}|s_{t}))],$$

Among them, $-\log(\pi (a_{t}^{*}|s_{t}))$ represents the negative log-likelihood loss of performing the optimal action $a_{t}^{*}$ in state, which is used to measure the difference between the prediction of the policy network in the target state and the actual optimal action.

### 2.3. Experimental Setup

#### 2.3.1. Candidate RL Algorithms

To comprehensively evaluate the performance of the hybrid closed-loop reinforcement learning algorithm with a safety controller proposed in this paper, we compare it with three advanced reinforcement learning (RL) algorithms widely used in continuous control tasks—Soft Actor–Critic (SAC), Proximal Policy Optimization (PPO), and Deep Deterministic Policy Gradient (DDPG) (their core principles and specific implementation details have been elaborated in Section 2.2). To ensure the fairness of the experiments, except for the distinctive structures unique to each algorithm (such as the safety controller module of the proposed algorithm, the entropy maximization strategy of SAC, and the clipped objective function of PPO), the rest of the key network structures and training parameters are strictly kept consistent, such as the number of layers and hidden units of the LSTM in the feature extraction layer, and the parameters of the fully connected layers of the Actor and Critic networks. During the training process, to adapt to the individual metabolic heterogeneity of simulated subjects, such as differences in insulin sensitivity and carbohydrate absorption rate, we train a separate model for each subject. All candidate algorithms are trained through 400,000 steps of environment interaction for each of the 3 different random seeds. After each training iteration (10 days), 20 evaluation simulations (each lasting 1 day) are conducted to monitor the convergence trend (fluctuation of the reward value) and early performance (incidence of hypoglycemia) of the algorithm. After the training is completed, 100 independent evaluation simulations are carried out for each subject (each simulation lasts for 1 day, a total of 288 steps, with a decision step every 5 min). The simulation scenarios cover real-life disturbances such as meal time deviation (±15 min) and carbohydrate measurement error (±10%) to comprehensively verify the performance of the algorithm in blood glucose control accuracy and safety robustness.

#### 2.3.2. Clinical Reference Method

In this paper, the ideal basal-bolus insulin treatment regimen (Basal-Bolus Insulin, BBI) is selected as the clinical benchmark algorithm. This regimen is the most commonly used, classic, and large-scale clinically verified insulin treatment strategy in current diabetes management. Due to its high recognition among clinicians, it is listed as the gold standard for evaluating the performance of insulin bolus algorithms in the “American Diabetes Association (ADA) Guidelines” [48]. Its core logic is the precise calculation of insulin dosage based on the patient’s individual characteristics. It requires patients or caregivers to inform the carbohydrate (CHO) content of future meals 20 min in advance so that the algorithm can dynamically adjust the insulin infusion strategy, which specifically consists of three parts: basal insulin (continuous low-dose infusion to maintain stable blood glucose during fasting and non-eating states), meal-time insulin bolus (rapid infusion before meals to offset the blood-glucose-raising effect of dietary carbohydrates), and corrective insulin (additional infusion to correct blood glucose levels when real-time blood glucose deviates from the target range). To ensure the standardization and fairness of the experiment, all virtual patients are simulated in a predefined typical three-meal-a-day scenario: breakfast is consumed at 7:00 (40 g of carbohydrates), lunch at 12:00 (80 g of carbohydrates), and dinner at 19:00 (60 g of carbohydrates). This dietary setting conforms to the regular dietary recommendations for adult patients with type 2 diabetes. The differences in meal times and carbohydrate contents can effectively simulate the impact of dietary structure fluctuations on blood glucose in real life, providing a unified perturbation scenario for subsequent comparison of the post-meal blood glucose control abilities of different algorithms.

The formula for calculating the insulin dosage delivered to the patient by BBI at time t is shown in Equation (18):

(18) $$I_{t}=BR+(c_{t}>0)\cdot (\frac{c_{t}}{CIR}+cool\cdot \frac{g_{t}-g_{target}}{ISF}),$$

Among them, $c_{t}$ is the estimated carbohydrate intake, which is manually entered by the patient or medical staff; $g_{t}$ is the currently monitored blood glucose level, while $g_{target}$ is the target blood glucose level to be controlled, usually 140 mg/dL; the basal insulin rate BR, the carbohydrate-to-insulin ratio CIR, and the insulin sensitivity factor ISF are calculated based on the total daily insulin (TDI) provided by the simulator for each subject, which are BR=0.48⋅TDI, $CIR=\frac{500}{TDI}$, and $ISF=\frac{1800}{TDI}$, respectively.

#### 2.3.3. Hyperparameter Setting

To select the optimal hyperparameter combination, a hyperparameter tuning technique called grid search is used. Grid search is an exhaustive search method that combines pre-defined different hyperparameters and then trains and evaluates each combination. Finally, the hyperparameter combination with the best performance is selected as the hyperparameter configuration for the optimal model. The main hyperparameter search ranges used in this experiment and the finally selected hyperparameters are shown in Table 1.

To optimize the stability and convergence efficiency of model training and prevent gradient explosion caused by an excessively high learning rate in the initial stage or getting stuck in local optima due to an excessively low learning rate in the later stage, this training adopted a combined learning rate strategy of linear warm-up and cosine annealing. The specific settings are as follows: The total number of training steps is 400,000 steps, among which the first 20,000 steps are the warm-up stage: the learning rate linearly increases from an initially extremely small value to a preset target learning rate. The core function of this stage is to allow the model to gradually adapt to the data distribution in the initial training stage, preventing the initial state of parameters from being disrupted by a sudden high learning rate and laying a foundation for subsequent stable convergence. After the warm-up stage ends, it enters the cosine annealing decay stage: the learning rate starts from the target learning rate and decays with a decay coefficient of 0.998 until it reaches the minimum learning rate threshold, which is 0.01 of the target learning rate. The advantage of cosine annealing is that it can allow the model sufficient time to finely adjust parameters through slow and smooth decay in the later training stage, preventing convergence stagnation caused by a sudden drop in the learning rate.

#### 2.3.4. Performance Indicators

This paper will evaluate the performance of the proposed algorithm from two perspectives: reinforcement learning indicators and clinical indicators.

RL indicators: The goal of reinforcement learning is to maximize the cumulative reward. Therefore, the cumulative rewards obtained by the algorithm proposed in this paper in the simulation environment are statistically analyzed. During the simulation process, a penalty of −15 is given and the simulation is terminated for catastrophic hypoglycemic events (bg < 40 mg/dL) that endanger the patient’s life, while the blood glucose values in the remaining range are calculated according to the reward function discussed in Section 3.1. The reward for each step is between 0 and 1, and the maximum number of simulation steps is 288 (one day). Therefore, the range of the cumulative reward is [−15, 288].

Clinical indicators: The main goal of blood glucose control is to maintain the blood glucose level within the normal range and minimize the time that the blood glucose stays in the risk area. Therefore, the percentage of time within different blood glucose ranges is statistically analyzed, and the blood glucose risk index is calculated. The blood glucose risk index includes the overall blood glucose risk index, the hyperglycemia risk index, and the hypoglycemia risk index. A larger value of the risk index indicates that hypoglycemia or hyperglycemia occurs more frequently or is more severe. Table 2 summarizes the indicators used to evaluate the clinical performance of the model.

### 2.4. Simulated Patients

The experiment in this paper was carried out based on a simulated clinical environment, which is a crucial pre-step before human clinical research. The study used Simglucose, an open-source Python implementation of the UVA/Padova type 1 diabetes (T1D) simulator, to accurately simulate the dynamic changes in blood glucose levels of T1D patients. The UVA/Padova T1D simulator is currently the only computer simulation model for diabetes patients approved by the U.S. Food and Drug Administration (FDA). It can replace pre-clinical animal tests for new treatment strategies for type 1 diabetes and has extremely high clinical relevance and reliability. This simulator includes 30 computer-simulated subjects, covering three age groups: adults, adolescents, and children (10 in each group). At the same time, it integrates physical models of commercially available insulin pumps and glucose sensors, can simulate the measurement errors of the selected devices (such as sensor drift and pump infusion delay), and supports customized meal plans to simulate real-life eating scenarios. In this experiment, 10 adult and 10 child simulated patients were selected as the research subjects, and the Insulet insulin pump and GuardianRT glucose sensor models built into the simulator were used to ensure that the experimental conditions were highly consistent with clinical practical applications.

### 2.5. Experimental Environment

All the experiments in this paper were conducted on the AutoDL cloud server, and the detailed configuration is shown in Table 3.

## 3. Results and Discussion

### 3.1. Comparison of RL Candidate Algorithms

#### 3.1.1. Comparison of Cumulative Rewards (RL Candidates)

**Figure 8:**
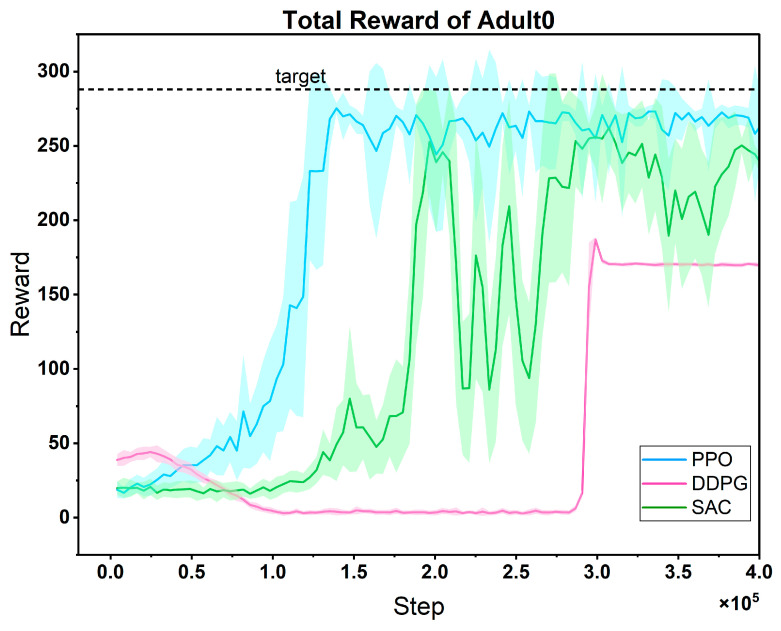
Comparison of reward curves of three RL candidate algorithms on the same patient. Light-shaded regions surrounding the mean reward curves (mean ± std); dashed line: maximum reward target (consistent in subsequent figures).

The cumulative reward training curves of three candidate algorithms in the task of optimizing the treatment strategy for the same patient (Figure 8) clearly show the performance differences among them:

In the early stage, the reward curve of PPO fluctuates greatly, which reflects the dynamic adjustment of the algorithm between exploration and exploitation. It actively tries different treatment plans through a stochastic strategy. As the training progresses, the fluctuation gradually decreases, and the reward value rises rapidly and remains stable. This is because its clipped probability ratio mechanism effectively limits the amplitude of policy updates, achieving a balance between exploration and exploitation, and finally reaching a high convergence value and strong stability. In contrast, although the reward curve of DDPG fluctuates less, its convergence speed lags significantly, and the final reward value is significantly lower than that of PPO. This is due to the inherent defect of its deterministic strategy—the lack of sufficient stochastic exploration makes it difficult to cover the potential optimal strategy space for patient treatment, and it is easy to fall into local optima. The performance of SAC features more “oscillation costs”: its reward curve fluctuates violently in the early and middle stages, with the slowest convergence speed. Although the reward value recovers in the later stage, it is still significantly lower than that of PPO. The core problem lies in the dynamic trade-off of entropy regularization. To maintain the stochasticity of the strategy, the adaptive adjustment of the temperature coefficient α easily leads to a strategy that is either too random (resulting in a sharp drop in rewards) or loses exploration ability (falling into local optima), increasing the difficulty of parameter tuning and intensifying the curve fluctuations.

The advantage of PPO is precisely that it avoids the above-mentioned defects: it solves the problem of “insufficient exploration” in DDPG through approximate trust region optimization, covering the potential optimal space for patient treatment with a more active strategy search; it alleviates the problem of “severe fluctuations” in SAC through the clipping mechanism, achieving more stable policy updates; at the same time, its convergence speed is much faster than that of DDPG and SAC, which is more suitable for the actual need of “rapid decision-making in patient treatment”. In summary, the high performance (high convergence value), strong stability (low late-stage fluctuations), and fast convergence speed (finding effective strategies in a short time) demonstrated by PPO in this task make it an ideal choice for further research. It can continue to optimize personalized treatment strategies and cope with the dynamic changes in patients’ conditions, showing greater potential for clinical application.

#### 3.1.2. Comparison of Clinical Indicators (RL Candidates)

Table 4 lists the comparison of patients’ blood glucose levels in each risk level range among three candidate RL algorithms in 100 evaluation simulations. Starting from the core goal of diabetes management-maintaining blood glucose homeostasis and avoiding extreme blood glucose risks—the performance differences among the three RL algorithms directly reflect their clinical applicability. Although the SAC algorithm can effectively control hypoglycemia, the proportion of hyperglycemia is as high as 47.22% (TAR1), which means that patients are exposed to a hyperglycemic environment for a long time, increasing the risks of chronic complications such as nephropathy and retinopathy, revealing its insufficiency in the hyperglycemia regulation strategy. The DDPG algorithm can control hypoglycemia more thoroughly, but the time in range (TIR) is only 54.92%, and the proportion of hyperglycemia still reaches 42.23% (TAR1). It may be due to the insufficient exploration of the deterministic strategy, failing to cover better hyperglycemia control schemes, resulting in limited overall blood glucose management effects. The PPO algorithm perfectly fits the ideal state of diabetes management: its time in range (TIR) is as high as 76.07%, which means that patients’ blood glucose is within the safe range (70–180 mg/dL) for nearly 80% of the day, which is crucial for reducing blood glucose fluctuations, protecting islet function, and reducing the risks of complications. Meanwhile, the proportion of severe hypoglycemia (TBR2) is only 0.07%, and the proportion of severe hyperglycemia (TAR2) is as low as 0.23%, avoiding the risks of extreme blood glucose events and achieving a balance between “safety” and “effectiveness”, indicating that PPO can more evenly handle the three major goals of “avoiding hypoglycemia”, “maintaining normal blood glucose”, and “controlling hyperglycemia” without sacrificing one for the other.

### 3.2. Comparison of Reward Functions

**Figure 9:**
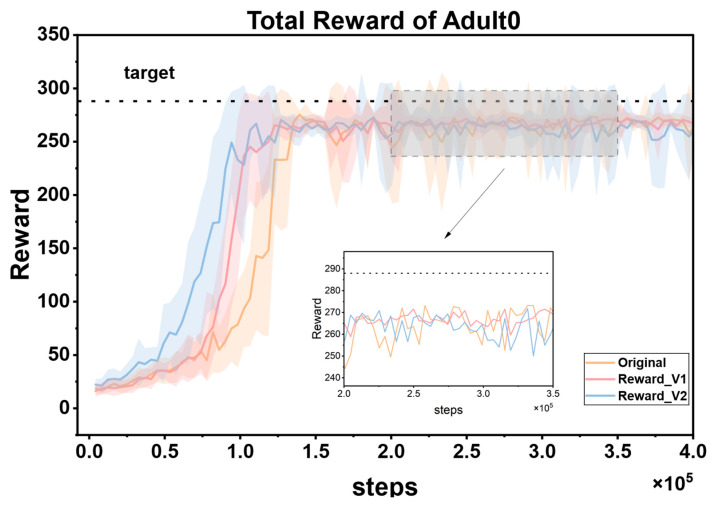
Comparison of cumulative reward curves of three reward functions for the same patient.

To fully verify the superiority of the proposed reward function, this study designed multiple groups of comparative experiments. First, it was directly compared with the reward mechanism in Reference [45]. Second, a simplified hypoglycemia penalty strategy was additionally introduced as a baseline; that is, the reward values in the range of 40–70 mg/dL were directly multiplied by a linear decay coefficient of 0.5. Under the framework of the PPO algorithm, three reward functions (the method in Reference [45], the linear decay baseline, and the proposed cubic spline smoothing strategy) were independently trained on the same patient dataset and repeated three times. During the experiment, all hyperparameters (such as learning rate, number of iterations, and network structure) and environmental configurations were strictly kept consistent. Figure 9 shows the dynamic changes of the three reward functions during the training process, namely Original (from Reference [45]), Reward_v1 (using the linear decay baseline), and Reward_v2 (the cubic spline smoothing strategy proposed in this paper). The reward curves corresponding to these three reward functions show obvious differences.

The cumulative reward curve reflects the overall rewards obtained by the reinforcement learning agent during the training process and can intuitively show the learning effect of the agent and the quality of the strategy. As can be seen from the figure, Reward_v2 performs the best. Its reward curve converges faster, which means that the agent can approach the optimal strategy in fewer training steps, greatly improving the training efficiency. At the same time, the curve maintains low volatility throughout and stably approaches the target reward value, indicating that the agent’s strategy performance is more stable, without significant fluctuations, and can continuously and reliably obtain higher rewards. In contrast, the curve of the Original reward function oscillates significantly, which may cause the agent to be affected by unstable reward signals during the learning process, making it difficult to optimize the strategy stably. It is easy to fall into the dilemma of strategy oscillation or local optimum and fail to find the global optimal strategy in time, thus reducing the training efficiency and final performance. The effect of Reward_v1 is at an intermediate level. Although it performs better than Original, there is still a certain gap in terms of convergence speed and stability compared with Reward_v2.

The cubic spline smoothing strategy proposed in this paper has significant advantages as a reward function. By constructing a non-linear continuous function, it effectively eliminates the mutation points in the reward signal. On the one hand, this characteristic can provide smooth policy gradient feedback for the PPO algorithm. In reinforcement learning, the smoothness of policy gradient feedback is crucial for the policy update of the agent. A mutated reward signal may lead to biases in policy gradient calculation, which in turn causes policy oscillations or getting stuck in local optima. However, Reward_v2 avoids these problems. On the other hand, through fine-grained gradient design, it can guide the agent to quickly capture the optimal policy during the exploration of the environment. When facing a complex environment, the agent can more accurately adjust its behavior based on the smooth and effective reward signal, so as to find the policy that maximizes the cumulative reward more quickly during the training process. Ultimately, it achieves a dual improvement in training efficiency and convergence stability.

### 3.3. Comparison of Feature Extraction Modules

As the core driving unit of the MPC framework, the prediction accuracy of the blood glucose prediction module directly determines the decision-making quality and safety of the entire closed-loop control system. Specifically, the earlier the prediction error of the blood glucose prediction module converges to a threshold below 15 mg/dL, the earlier the MPC controller can initiate the multi-step simulation and optimization process. This means that the system can impose effective constraints and refined adjustments on the original action output of the DRL agent earlier, prompting the DRL agent to learn the optimal strategy in a safer exploration space, thereby shortening the time window during which patients are exposed to potential blood glucose fluctuation risks. Meanwhile, the lower the amplitude of the prediction error and the smaller the oscillation of the blood glucose prediction module, the stronger the robustness of the control sequence generated by the MPC based on the prediction trajectory, and the higher the reliability of the optimized actions output, ultimately improving the control stability of the overall system.

**Figure 10:**
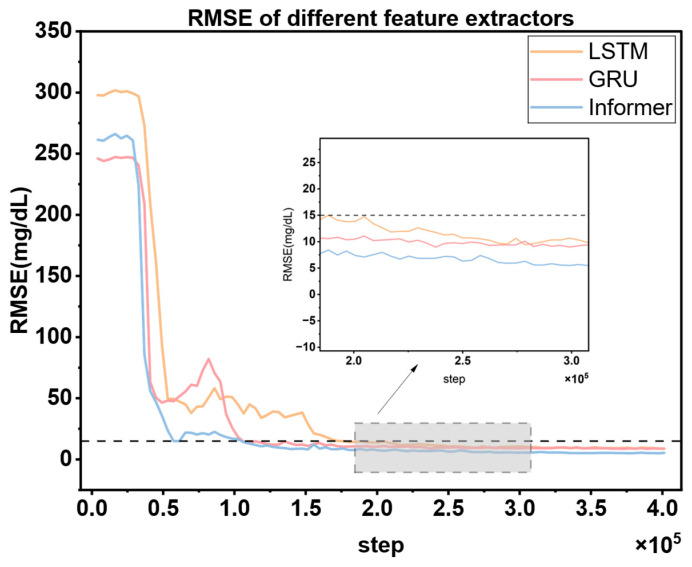
Comparison of RMSE training curves of different feature extractors under the same experimental conditions. The dashed line denotes the target RMSE value (15 mg/dL) for the glucose prediction module.

To verify the superiority of the Informer module as the feature extraction unit of the blood glucose prediction module, this paper designs a comparative experiment: Three typical time-series feature extraction modules, namely GRU, LSTM, and Informer, are selected and independently trained on the same patient dataset under the same algorithm framework (each group of experiments is repeated three times to ensure the robustness of the results). Except for the feature extraction module, the rest of the network architecture, such as input dimensions, hidden-layer parameters, optimizer configuration, etc., and hyperparameters are strictly kept consistent. During the experiment, the dynamic changes of the prediction errors of each model are continuously recorded, and the error trend curves are plotted (as shown in Figure 10) to evaluate the impact of different feature extraction modules on blood glucose prediction performance.

Figure 10 shows that in the blood glucose prediction task, the Informer feature extraction module exhibits obvious advantages over GRU and LSTM. Its prediction error converges the fastest and can first drop below the safety threshold of 15 mg/dL, enabling the MPC controller to initiate the multi-step simulation and optimization process in advance. This effectively shortens the time window during which patients are exposed to potential blood glucose fluctuation risks. Meanwhile, the amplitude of the prediction error of Informer is the lowest and its oscillation range is the smallest. This means that the MPC can output a more robust control sequence based on the prediction trajectory generated by Informer, and the reliability of the optimization actions is significantly improved. Ultimately, the control stability of the overall system is enhanced. The experimental results fully verify the superiority of Informer as the feature extraction unit of the blood glucose prediction module.

### 3.4. Comparison Between PPO and Hybrid Control Strategies PPO + MPC

Through the previous comparative experiments, we selected PPO as the RL algorithm for our proposed hybrid control model. To further improve the convergence speed of the PPO algorithm and reduce the number of unsafe interactions between the RL Agent and patients, we introduced a stage of MPC prediction simulation to simulate multiple trajectories of the actions output by RL and select the action with the highest cumulative reward as the optimal action for RL to fine-tune the RL Agent. The specific implementation details have been elaborated in Section 2.3.

To verify the effectiveness of our proposed hybrid control strategy, we conducted training and evaluation on 10 adult simulated patients and 10 child simulated patients. After the training was completed, 100 one-day evaluation simulations were still carried out. The results are as follows.

#### 3.4.1. Comparison of Cumulative Rewards (PPO vs. PPO + MPC)

**Figure 11:**
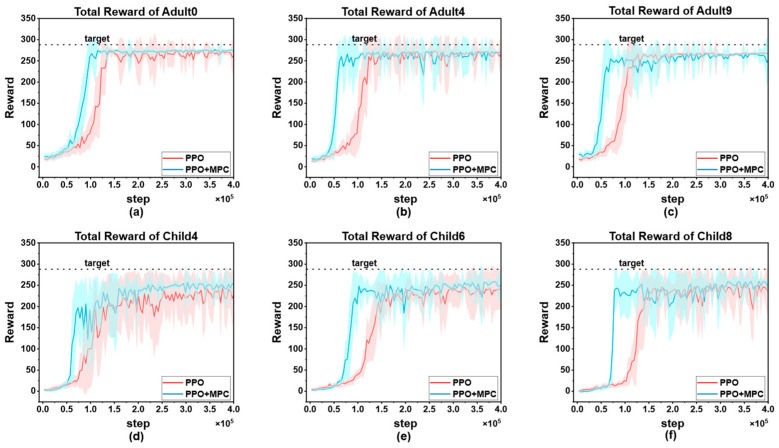
Comparison of cumulative reward curves: (**a**) cumulative reward curves of Adult0; (**b**) cumulative reward curves of Adult4; (**c**) cumulative reward curves of Adult9; (**d**) cumulative reward curves of Child4; (**e**) cumulative reward curves of Child6; (**f**) cumulative reward curves of Child9.

To visually demonstrate the improvement of the proposed algorithm on the convergence speed of PPO, we selected the cumulative reward curves of 3 patients from each of the adult group and the child group for comparative display. As shown in Figure 11.

In both adult and child patients, it can be observed that the convergence speed of the hybrid control strategy combining PPO and MPC is significantly faster than that of the pure PPO strategy. The core reason for this difference lies in the fact that during the prediction and simulation phase of MPC, by excluding dangerous actions and selecting the optimal action with the highest cumulative reward, it provides more accurate learning guidance for the RL agent, enabling it to master effective blood-glucose control strategies more quickly. This result is of great significance in clinical applications—the more interactions an RL agent has with a patient before learning a stable and effective strategy, the higher the probability of the patient experiencing dangerous conditions such as severe hypoglycemia. The hybrid strategy reduces the unsafe interactions between the agent and the patient through the “pre-screening” mechanism of MPC, and precisely provides a feasible solution to the key problem in the current clinical application of RL in blood-glucose control, that is, how to reduce patient risks in the early stage of learning. Overall, the advantages of the hybrid strategy are not only reflected in the improvement of convergence speed, but also in the balance between “learning efficiency” and “patient safety” through the complementarity of MPC and RL, providing a more robust path for the clinical translation of RL technology in blood-glucose management.

It is worth noting that although the hybrid strategy shows consistent advantages in the overall population, individual differences still result in a significantly larger 90% confidence interval for some patients (such as Child 4, Figure 11d) compared to other subjects. This phenomenon reflects the complexity of blood glucose control—there are natural differences in insulin sensitivity, metabolic rate, diet and exercise patterns among different patients, which may lead to local fluctuations during the algorithm learning process. However, the key finding is that even in the presence of individual differences, the hybrid strategy can achieve more effective control than the PPO strategy in each patient. It has a faster convergence speed, and the reliability of the final optimized actions is higher than that of the pure PPO strategy. This indicates that through the dynamic constraint mechanism of MPC, the hybrid strategy has a certain robustness buffering ability, which can maintain the stability of the core control objectives while adapting to individual physiological differences.

**Figure 12:**
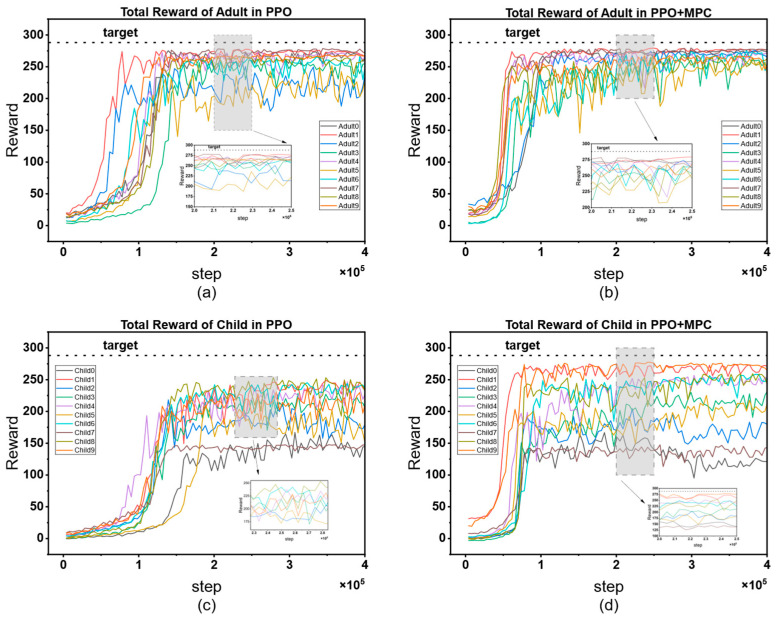
Comparison of cumulative reward curves between the adult group and the child group: (**a**) cumulative reward curve of the adult group in PPO; (**b**) PPO + MPC cumulative reward curve for the adult group; (**c**) PPO cumulative reward curve for the children’s group; (**d**) cumulative reward curve of the children’s group for PPO + MPC.

The cumulative reward curves of all patients are shown in Figure 12. By horizontally comparing the model performance between the adult group and the child group, it can be seen that the PPO strategy shows a slow convergence speed in both groups, and there are significant differences in the convergence speed among patients. There are also large fluctuations in the finally converged reward values. This phenomenon is mainly due to the individual differences in the physiological structures of patients, which lead to large differences in the time for the agent to learn effective strategies. In contrast, the PPO + MPC hybrid control strategy has a more consistent convergence speed in both groups, indicating that in the prediction fine-tuning stage, the optimal actions generated by MPC simulation can assist the agent to achieve more efficient and stable learning, and to some extent, alleviate the problem of fluctuations in the model convergence speed caused by the physiological differences among patients. Further analyzing from the longitudinal dimension, the performance of both the PPO and PPO + MPC hybrid strategies in the child group is significantly worse than that in the adult group. This is because the physiological parameters and blood glucose dynamics of children are more complex than those of adults. The model fails to fully capture the unique blood glucose change patterns of the child population, so it fails to learn effective control strategies for children.

Table 5 presents the average cumulative reward results of adult and child patients in 100 simulated evaluations. The data shows that the average cumulative reward of the PPO + MPC hybrid control strategy is significantly better than that of the pure PPO strategy in both groups, indicating that the long-term control effect of the system is effectively improved after the introduction of the MPC mechanism. To further verify the statistical significance of this difference, we conducted paired t-tests on the evaluation data of the two groups of patients, respectively: the results of the adult group show that the cumulative reward of the PPO + MPC strategy is significantly higher than that of the pure PPO (t = 4.1721, *p* = 1.6 × 10^−5^); in the child group, this improvement is even more significant (t = 4.6018, *p* = 2 × 10^−6^). The *p*-values of both groups are less than 0.05, suggesting that the introduction of MPC has statistical reliability in improving the cumulative reward.

**Figure 13:**
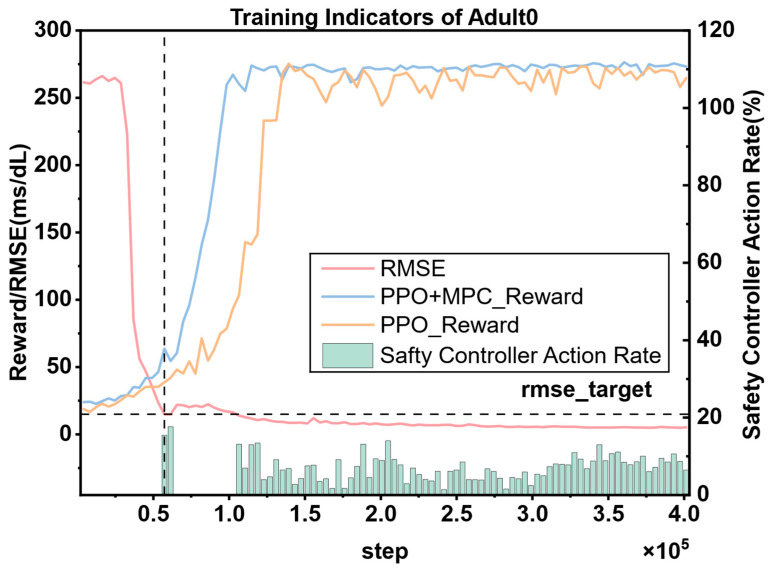
The cumulative reward curve, the prediction error change curve of the blood glucose prediction module, and the action ratio of the safety controller for the same patient. The vertical dashed line represents the step when the RMSE predicted by the blood glucose prediction model first drops to the target value (15 mg/dL).

To more intuitively demonstrate the interactions between various modules in the entire system, the cumulative reward curve of DRL, the change curve of the prediction error of the blood glucose prediction module, and the action frequency curve of the safety controller are plotted in the same figure (Figure 13). Meanwhile, the cumulative reward curve of the pure PPO algorithm is plotted for comparison. From the figure, it can be clearly observed that when the root-mean-square error (RMSE) of the blood glucose prediction module drops below 15 mg/dL, the system initiates the simulation based on MPC, and the safety controller starts to act immediately. The optimal actions output by the MPC controller and the safety controller are provided to the PPO model for learning. Under the action of this collaborative mechanism, the growth rate of the reward curve of PPO using the hybrid control strategy is significantly higher than that of the pure PPO reward curve, and it can converge faster. In addition, the reward value that the PPO model under the hybrid control strategy finally converges to is higher than that of the pure PPO strategy, and the curve is smoother with smaller fluctuations. This indicates that under the dual action of the MPC controller and the safety controller, not only can the learning speed of PPO be improved, but also the stability of its training can be enhanced. For diabetic patients, this stability is crucial because it helps to achieve more accurate and reliable blood glucose control, thereby improving the patients’ health management level and quality of life.

#### 3.4.2. Comparison of Clinical Indicators (PPO vs. PPO + MPC)

We also compared the proportions of patients’ blood glucose levels in each risk level range under the control of the PPO and PPO + MPC models. Meanwhile, to verify the effectiveness in clinical applications, we also compared the control performance of the two models with the gold standard of insulin bolus injection in clinical diabetes management, the BBI. The results are shown in the following Table 6.

It can be seen from the tabular data that the time-in-range (TIR) of patients’ blood glucose under the control of the PPO + MPC hybrid model is significantly higher than that under the pure MPC model and the clinical benchmark algorithm BBI. Meanwhile, the time-above-range for hyperglycemia (TAR1) and severe hyperglycemia (TAR2) are both significantly lower than those of the latter two. Although the time-below-range of this model is slightly higher than that of BBI (0.86% for BBI and 1.52% for PPO + MPC), the time-below-range for severe hypoglycemia is only 0.1%, which is on par with BBI (0.09%). Overall, our model outperforms the clinical benchmark in blood glucose control for adult patients. However, the results in the pediatric group show significant differences—the control effect of the clinical benchmark BBI is significantly better than that of the PPO + MPC and pure PPO models. This phenomenon is mainly due to the more complex physiological parameters (such as higher insulin sensitivity and greater fluctuations in metabolic rate) and blood glucose dynamics (such as steeper post-meal blood glucose peaks and higher risk of nocturnal hypoglycemia) of pediatric patients, which cause the RL model to fail to fully capture the personalized needs of the pediatric population.

The performance divergence between the children’s group and the adult group mainly stems from the multiple superimposed effects of the unique physiological characteristics of children, insufficient data support, and the lack of adaptability to clinical constraints. Physiologically, children’s insulin sensitivity is 30–50% higher than that of adults, and the daily blood-glucose fluctuation range is twice that of adults. Such high-frequency and drastic metabolic dynamic changes make it difficult for RL agents relying on fixed reward functions to accurately capture the laws of instantaneous fluctuations. Moreover, the fluctuations of hormones such as growth hormone during puberty have a significant antagonistic effect on insulin, further increasing the complexity of blood-glucose regulation. The limitations at the data level are also crucial. Children’s samples account for only 28% of the training data, and there is a lack of annotation information on pubertal hormone fluctuations. As a result, the model has difficulty fully learning the specific metabolic patterns of children, and its generalization ability is limited. In addition, children’s perception threshold for hypoglycemia is 15–20% lower than that of adults, but the current reward function does not separately optimize the hypoglycemia penalty weight for this characteristic, which may lead to a conservative control strategy and ultimately affect the control effect [49].

Despite the above challenges, the core advantages of the hybrid strategy are still evident in the children’s group. The gap between the Time in Range (TIR) and the baseline Basal-Bolus Index (BBI) can be compensated for through targeted optimization, such as introducing a module specific to children’s physiological characteristics and dynamically adjusting the weights of the reward function. This result suggests that the DRL+MPC collaborative framework of the PPO + MPC hybrid strategy has good scalability. In the future, through the design concept of a “general framework + population-specific module”, it is expected to achieve blood glucose control effects in children comparable to those in adults with this model.

It is worth noting that the control of BBI relies on meal announcements and manual input of carbohydrate intake. If the actual carbohydrate intake of the patient does not match the estimated value (e.g., snacks are not recorded), or the data is not input into the system in a timely manner, the control effect will decline. In addition, the total daily insulin dose (TDI) of the patient needs to be estimated by professional doctors based on indicators such as body weight and glycated hemoglobin (HbA1c). This process is susceptible to human errors and is also a key factor restricting the performance of BBI. In contrast, our RL algorithm uses a fully automatic closed-loop control. It only requires the historical data of the continuous glucose monitoring (CGM) sensor and the injection records of the insulin pump to independently decide the insulin infusion dose. This mode not only completely reduces the cognitive burden on patients (no need for manual input or meal announcements) but also avoids the interference of human factors such as carbohydrate estimation errors and TDI estimation deviations on the control performance, which better meets the needs of patients in real-world usage scenarios.

#### 3.4.3. Comparison Under Different Dietary Disturbances

**Figure 14:**
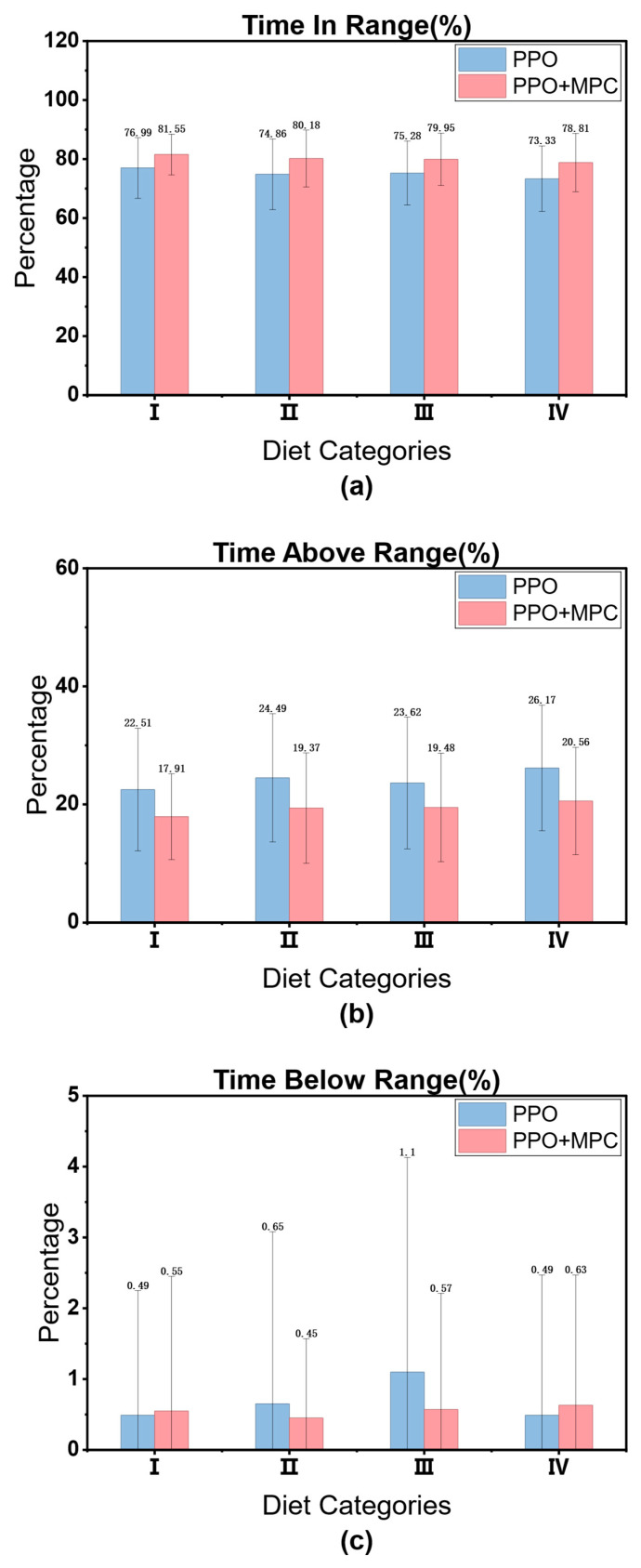
Time proportion of patients in each risk interval under different dietary scenarios: (**a**) Proportion of normal blood glucose time in different dietary scenarios; (**b**) Proportion of hyperglycemia time in different dietary scenarios; (**c**) Proportion of hypoglycemia time in different dietary scenarios. Note: The numerical values displayed at the top of each bar in subfigures (**a**–**c**) represent the mean time proportion of patients in each blood glucose risk category for each dietary scenario. I: Fixed diet; II: Fluctuating meal portion; III: Fluctuating meal time; IV: Random snacks.

To more comprehensively validate the applicability and robustness of the model in complex real-world dietary scenarios, that is, to simulate the diversity and uncertainty of patients’ daily dietary patterns, we selected four representative dietary scenarios: fixed diet (an ideal state where the time, portion size, and food types of three meals a day are strictly consistent), fluctuating meal portion (the portion size of each meal is randomly adjusted within the range of ±20–30% of the regular value, simulating the portion changes caused by patients’ appetite or food estimation errors), fluctuating meal time (the time of each meal deviates from the established plan by ±20–30%, reflecting the time delay/advance in scenarios such as overtime work and social activities), and random snacks (1–3 additional irregular snacks, such as fruits, nuts, or pastries, are added daily, simulating patients’ occasional snack intake). We conducted a systematic evaluation of the trained model using these scenarios. To ensure the statistical significance and reliability of the experimental results, each dietary scenario was repeated 100 times for independent 24-h blood glucose simulation evaluations. During the evaluation process, we focused on counting three core blood glucose control indicators: TIR, TBR, and TAR. Through the quantitative analysis of these indicators, we were able to clearly understand the performance differences of the model under different dietary interferences. The experimental results are shown in Figure 14 below.

It can be clearly seen from the figure that even when facing dietary disturbances, such as real-life scenarios with high meal variance, variable meal times, or a base meal plus random snacks, although the model performance shows slight fluctuations compared to the fixed-diet scenario, the core indicator TIR (the proportion of time that the patient’s blood glucose is within the normal range) still stably remains above the clinical standard of 70%. Moreover, the time proportions of hyperglycemia (TAR) and hypoglycemia (TBR) do not show a significant increase. This fully demonstrates that our model can still maintain a robust blood-glucose regulation ability when dealing with complex dietary disturbances, meeting the basic clinical requirement for “stability”. After all, in the real world, patients’ diets cannot be completely fixed, and this anti-disturbance ability is a key prerequisite for the model to be applied clinically. Compared with the pure PPO model, the PPO + MPC hybrid control strategy we proposed shows statistically significant advantages in the three key indicators of TIR, TBR, and TAR (As shown in Table 7). For example, in the “base meal + random snacks” scenario, the TIR of PPO is 73.33%, while that of the hybrid model is increased to 78.81%. The TBR is reduced from 26.17% of PPO to 20.56%, and TAR also shows similar improvements. This improvement stems from the complementarity of the two strategies: the reinforcement learning ability of PPO is responsible for capturing long-term blood-glucose change patterns, such as blood-glucose fluctuation trends under different dietary patterns, while the model predictive control of MPC enables real-time dynamic adjustment, such as dealing with the rapid blood-glucose rise after sudden snack intake. The combination of the two effectively solves the problem of “being easily affected by disturbances and having large performance fluctuations” of a single RL model, significantly enhancing the adaptability and reliability of the model.

## 4. Conclusions

This paper experimentally compares the performance of three advanced reinforcement learning (RL) algorithms, namely SAC (Soft Actor–Critic), DDPG (Deep Deterministic Policy Gradient), and PPO (Proximal Policy Optimization), in blood glucose management for patients with type 1 diabetes (T1D). Due to the inherent drawbacks of SAC and DDPG (although SAC can ensure the entropy regularization of the policy to enhance exploration ability, its sample efficiency is low, making it difficult to converge quickly in limited patient interactions; DDPG is highly sensitive to hyperparameters, and the training process is prone to divergence, resulting in large fluctuations in blood glucose control and even dangerous hypoglycemic/hyperglycemic events), their performance in terms of cumulative rewards (reflecting long-term blood glucose control effects) and core clinical indicators (TIR: Time in Range; TBR: Time Below Range; TAR: Time Above Range) is significantly lower than that of PPO. PPO limits the policy update amplitude through the clip ratio mechanism, which not only ensures training stability but also maintains high sample efficiency. It is more suitable for handling continuous, high-dimensional control tasks with extremely high safety requirements such as blood glucose management. Therefore, it is selected as the core RL algorithm for the proposed hybrid control strategy.

To address the clinical translation bottlenecks of the pure RL model (long-term trial-and-error risks due to slow convergence and potential harm to patients from unsafe actions), we propose a hybrid strategy of PPO + MPC (Model Predictive Control):

Data recycling: Collect the blood glucose-action interaction data generated during the PPO training process to train a blood glucose prediction model (capturing individual patient blood glucose dynamics, such as insulin action delay and diet absorption rate).

MPC prediction and simulation: Use this prediction model as the patient dynamic model for MPC and introduce a “prediction and simulation phase”. For the original actions output by the RL Agent, predict the blood glucose changes in the next 30 min through MPC, filter out dangerous actions that may lead to hypoglycemia or hyperglycemia, and fine-tune the safe actions.

Dual safety guarantee: In addition to the action screening by MPC, add a safety controller based on clinical rules to form a three-layer safety mechanism of “RL strategy learning+MPC short-term prediction + safety controller backup”, effectively reducing short-term blood glucose risks.

The experimental results show that the hybrid strategy significantly improves the convergence speed of PPO, which means a substantial reduction in the number of trial-and-error interactions with patients. At the same time, it increases the TIR from 69.97% of the original PPO to 72.51% (*p* < 0.05). This performance is not only better than that of pure PPO but also exceeds the 69.85% of the clinical gold-standard BBI, demonstrating better clinical safety. However, in the group of children with T1D, the hybrid strategy fails to form an effective strategy, and all performance indicators are lower than those of BBI. It is speculated that the reason may be that the blood glucose dynamics in children are more complex, and the existing blood glucose prediction models are not optimized for the individual characteristics of children, resulting in a decrease in the prediction accuracy of MPC and the inability to effectively assist the fine-tuning of RL actions. This result suggests that in the future, more accurate blood glucose prediction models and adaptive control strategies need to be designed according to the physiological characteristics of the child population.

Finally, to simulate dietary disturbances in real life, we introduced different dietary disturbance scenarios in an adult patient. The results showed that the Time in Range (TIR) of the hybrid strategy still remained above the clinical standard of 70%, and there was no significant increase in Time Below Range (TBR) and Time Above Range (TAR). This indicates that the model still has robust blood glucose control ability under complex dietary disturbances, solving the problem of the pure Reinforcement Learning (RL) model being “easily affected by environmental changes” and better meeting the requirements of clinical practical applications.

More importantly, this work provides important empirical support and framework reference for the practical application of Reinforcement Learning (RL) technology in clinical blood glucose management:

Solving the “robustness bottleneck” of RL clinical translation: In the real clinical environment, factors such as patients’ diet, exercise, and emotions are highly uncertain. Although traditional RL models can learn autonomously, they often perform poorly when facing unseen disturbances. Our hybrid strategy combines the “long-term learning advantage” of RL with the “short-term predictive control” of Model Predictive Control (MPC), enabling the model to both “remember” long-term patterns and “cope with” sudden changes, providing a feasible solution for the stable application of RL technology in clinical practice.

Validating the clinical value of RL in blood glucose management: The model can still maintain performance that meets clinical standards under disturbance scenarios, indicating that RL can not only “simulate” blood glucose control in the laboratory environment but also “effectively” control blood glucose in scenarios close to real life. This lays the foundation for the subsequent translation of RL technology into clinical practice. In the future, it is expected to become an intelligent auxiliary tool for clinicians, helping doctors adjust insulin pump parameters more accurately or providing personalized diet and exercise advice for patients.

Providing directions for future research: Our work demonstrates the potential of the integration of “RL + traditional control strategies”. In the future, more complex hybrid models (such as blood glucose prediction modules combined with deep learning) can be further explored, or it can be extended to a wider range of clinical scenarios (such as diabetic patients with complications) to further improve the clinical applicability of RL technology.

In short, our research not only experimentally verifies the effectiveness of the hybrid model but also builds a bridge for RL technology to move from the “laboratory” to “clinical practice”. It is expected to bring more intelligent and reliable solutions for blood glucose level management in diabetic patients, ultimately improving their quality of life.

## Figures and Tables

**Table 1:** Hyperparameter selection.

Hyperparameter	Search Scope	Final Choice
Batch size	256, 512, 1024	1024
LSTM layers	1, 2, 3	2
LSTM hidden units	8, 16, 32	16
Historical CGM steps	6, 12, 24	12
Learning rate	1 × 10^−4^, 3 × 10^−4^, 5 × 10^−4^, 1 × 10^−3^	3 × 10^−4^
Encoder layers	1, 2, 3	1
Attention heads	4, 8, 16	4
Embedding vector dimension	32, 64, 128	64
Hidden layers of feedforward neural network	32, 64, 128	128

**Table 2:** Clinical performance evaluation indicators.

Risk Level	Symbolic Notation	Scope
Severe hypoglycemia	TBR-2	<54 mg/dL
Hypoglycemia	TBR-1	54–70 mg/dL
Normal blood sugar	TIR	70–180 mg/dL
Hyperglycemia	TAR-1	180–250 mg/dL
Severe hyperglycemia	TAR-2	>250 mg/dL

**Table 3:** Experimental environment configuration.

Environment	Version
Miniconda	conda3
Python	3.10
Cuda	11.3
GPU	RTX 4090 (24 GB)
CPU	16 vCPU Intel(R) Xeon(R) Gold 6430
Deep learning framework Reinforcement learning library	Pytorch 1.26.4 gym 0.9.4

**Table 4:** Comparison of candidate RL clinical indicators (%).

RL Algorithm	TBR2	TBR1	TIR	TAR1	TAR2	Reward
SAC	0.0	0.05	34.62	47.22	18.11	237.24
DDPG	0.0	0.0	54.92	42.23	2.85	259.80
PPO	0.07	0.47	76.07	23.16	0.23	273.06

**Table 5:** Comparison of the average cumulative rewards of all patients in 100 evaluation simulations.

Patients ID	PPO	PPO + MPC	Patients ID	PPO	PPO + MPC
Adult#00	273.06	274.37	Child#00	222.37	225.36
Adult#01	274.1	276.37	Child#01	243.04	242.97
Adult#02	262.48	265.57	Child#02	225.73	226.49
Adult#03	259.37	267.82	Child#03	232.06	236.05
Adult#04	270.97	270.7	Child#04	247.64	252.67
Adult#05	256.16	259.08	Child#05	209.62	227.4
Adult#06	269.65	271.77	Child#06	257.15	256.79
Adult#07	277.86	276.39	Child#07	241.22	246.43
Adult#08	261.62	266.64	Child#08	258.12	258.48
Adult#09	267.36	266.09	Child#09	233.74	238.44
AVG	267.26 ± 8.82	269.48 ± 7.1	AVG	237.09 ± 15.61	241.11 ± 13.01

**Table 6:** Proportions (%) of each risk interval for RL algorithms and clinical standards.

Method	TBR2	TBR1	TIR	TAR1	TAR2
Adult					
BBI	0.09	0.86	69.85	27.67	5.01
PPO	0.11	1.32	69.97	25.75	2.85
PPO + MPC	0.1	1.52	72.51	22.24	1.63
Child					
BBI	1.94	6.01	66.47	20.56	4.62
PPO	0.29	3.22	53.17	25.39	17.93
PPO + MPC	0.19	3.02	55.21	25.83	15.76

**Table 7:** Comparison of the significance (*p*-value) of each index under different dietary scenarios.

Dietary Scenarios	TBR	TIR	TAR
Fixed diet	0.0017 **	0.0093 **	0.0025 **
Fluctuating meal portion	0.0312 *	0.0087 **	0.0062 **
Fluctuating meal time	0.0205 *	0.0038 **	0.0146 *
Random snacks	0.0004 **	0.0011 **	0.0007 **

* indicates *p* < 0.05; ** indicates *p* < 0.01.

## Data Availability

The Simglucose dataset used in this study is publicly available at: https://github.com/jxx123/simglucose (accessed on accessed on 3 November 2024).
